# Supplementary material for: The causal relationship between the human gut microbiota and pyogenic arthritis: a Mendelian randomization study
Source: Front Cell Infect Microbiol. 2024 Nov 26;14:1452480. doi: 10.3389/fcimb.2024.1452480 (PMC11629706; doi:10.3389/fcimb.2024.1452480)
Supplement: Supplementary file 1 [file DataSheet1.zip › mendelian test/ebi-a-GCST90027482.csv_leave_one_out.pdf]

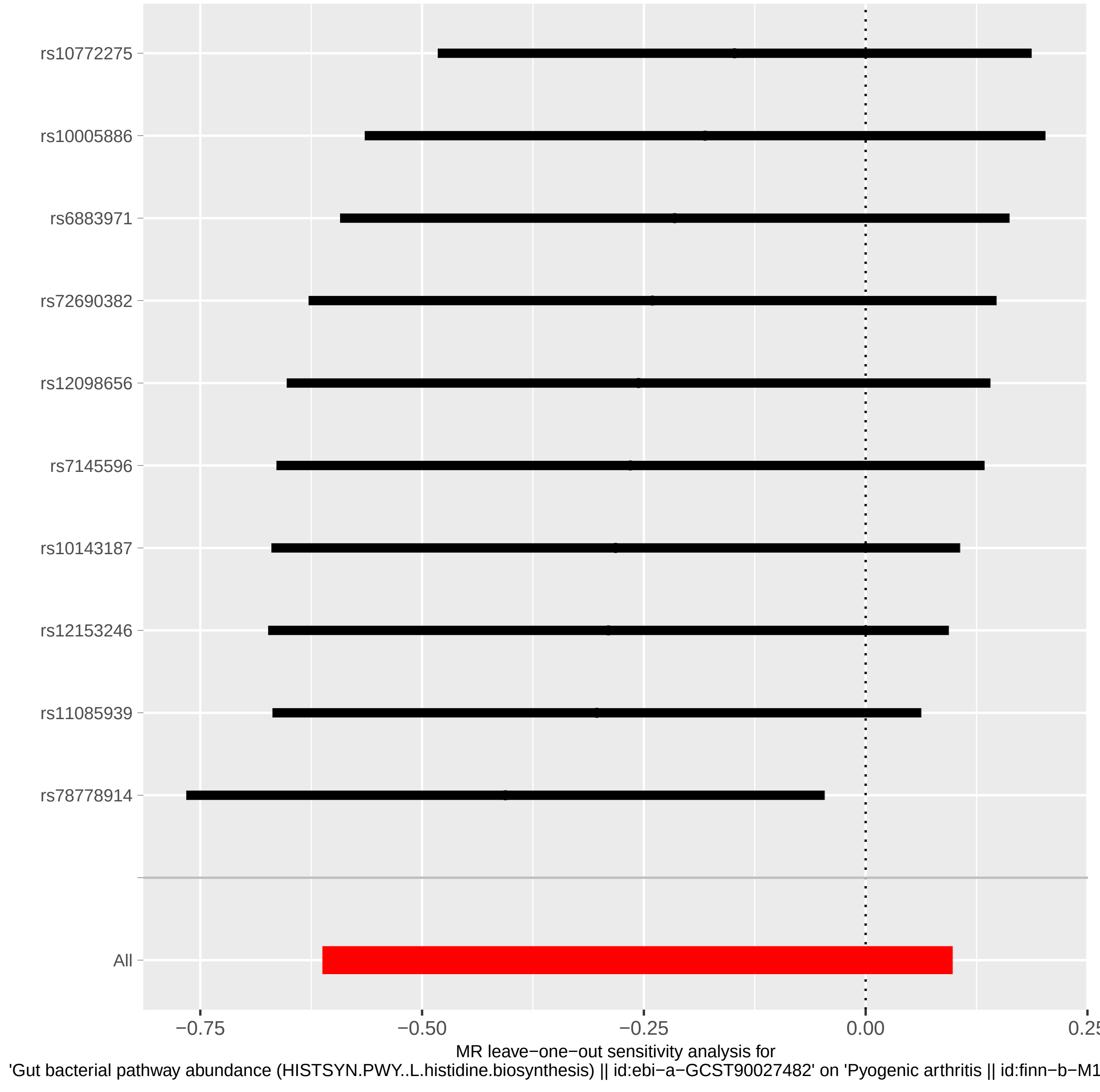

rs10772275
rs10005886
rs6883971
rs72690382
rs12098656
rs7145596
rs10143187
rs12153246
rs11085939
rs78778914
All
−0.75
−0.50
−0.25
0.00
0.25
MR leave−one−out sensitivity analysis for
'Gut bacterial pathway abundance (HISTSYN.PWY..L.histidine.biosynthesis) || id:ebi−a−GCST90027482' on 'Pyogenic arthritis || id:finn−b−M1
